# Sex differences in cognitive functioning of patients at-risk for psychosis and healthy controls: Results from the European Gene–Environment Interactions study

**DOI:** 10.1192/j.eurpsy.2019.10

**Published:** 2020-03-13

**Authors:** Stephanie Menghini-Müller, Erich Studerus, Sarah Ittig, Lucia R. Valmaggia, Matthew J. Kempton, Mark van der Gaag, Lieuwe de Haan, Barnaby Nelson, Rodrigo A. Bressan, Neus Barrantes-Vidal, Célia Jantac, Merete Nordentoft, Stephan Ruhrmann, Garbiele Sachs, Bart P. Rutten, Jim van Os, Anita Riecher-Rössler, the EU-GEI High Risk Study Group

**Affiliations:** 1 University of Basel, Department of Psychiatry, Basel, Switzerland; 2 Department of Psychology, Division of Personality and Developmental Psychology, University of Basel, Basel, Switzerland; 3 Department of Psychology, Institute of Psychiatry, Psychology & Neuroscience, King’s College London, London, United Kingdom; 4 Department of Psychosis Studies, Institute of Psychiatry, Psychology & Neuroscience, King’s College London, London, United Kingdom; 5 Faculty of Behavioural and Movement Sciences, Department of Clinical Psychology and EMGO+ Institute for Health Care Research, VU University, Amsterdam, The Netherlands; 6 Department of Psychosis Research, Parnassia Psychiatric Institute, The Hague, The Netherlands; 7 Department Early Psychosis, AMC, Academic Psychiatric Centre, Amsterdam, The Netherlands; 8 Mental Health Institute, Arkin, Amsterdam, The Netherlands, Amsterdam, The Netherlands; 9 Centre for Youth Mental Health, University of Melbourne, Melbourne, Australia; 10 LiNC—Lab Interdisciplinar Neurociências Clínicas, Depto Psiquiatria, Escola Paulista de Medicina, Universidade Federal de São Paulo—UNIFESP, São Paulo, Brazil; 11 Departament de Psicologia Clínica I de la Salut (Universitat Autònoma de Barcelona), Fundació Sanitària Sant Pere Claver (Spain), Spanish Mental Health Research Network (CIBERSAM), Barcelona, Spain; 12 University Paris Descartes, Hôpital Sainte-Anne, C’JAAD, Service Hospitalo-Universitaire, Inserm U894, Institut de Psychiatrie (CNRS 3557), Paris, France; 13 Mental Health Center Copenhagen, Copenhagen, Denmark; 14 Institute for Clinical Medicine, Faculty of Health Science, University of Copenhagen, Copenhagen, Denmark; 15 Department of Psychiatry and Psychotherapy, University of Cologne, Cologne, Germany; 16 Department of Psychiatry and Psychotherapy, Medical University of Vienna, Vienna, Austria; 17 Department of Psychiatry and Neuropsychology, School for Mental Health and Neuroscience, Maastricht, The Netherlands; 18 Department Psychiatry, Brain Centre Rudolf Magnus, Utrecht University Medical Centre, Utrecht, The Netherlands; 19 Department of Psychiatry and Psychology, School for Mental Health and Neuroscience (MHeNS), Maastricht University Medical Centre, Maastricht, The Netherlands; 20 King's College London, King's Health Partners Department of Psychosis Studies, Institute of Psychiatry, London, United Kingdom; 21 A full list of authors and affiliations appears in the Appendix.

**Keywords:** clinical high-risk, cognition, gender differences, risk for psychosis

## Abstract

**Background.:**

Sex differences in cognitive functioning have long been recognized in schizophrenia patients and healthy controls (HC). However, few studies have focused on patients with an at-risk mental state (ARMS) for psychosis. Thus, the aim of the present study was to investigate sex differences in neurocognitive performance in ARMS patients compared with HC.

**Methods.:**

The data analyzed in this study were collected within the multicenter European Gene–Environment Interactions study (11 centers). A total of 343 ARMS patients (158 women) and 67 HC subjects (33 women) were included. All participants completed a comprehensive neurocognitive battery. Linear mixed effects models were used to explore whether sex differences in cognitive functioning were present in the total group (main effect of sex) and whether sex differences were different for HC and ARMS (interaction between sex and group).

**Results.:**

Women performed better in social cognition, speed of processing, and verbal learning than men regardless of whether they were ARMS or HC. However, only differences in speed of processing and verbal learning remained significant after correction for multiple testing. Additionally, ARMS patients displayed alterations in attention, current IQ, speed of processing, verbal learning, and working memory compared with HC.

**Conclusions.:**

Findings indicate that sex differences in cognitive functioning in ARMS are similar to those seen between healthy men and women. Thus, it appears that sex differences in cognitive performance may not be specific for ARMS, a finding resembling that in patients with schizophrenic psychoses.

## Introduction

Sex differences in schizophrenia have been described in almost all features of the illness, including incidence, prevalence, age at onset, symptomatology, course, and in the response to treatment, but only reliably established in age at onset and course [1]. Sex-related differences in the illness course might be at least partially mediated by sex-related differences in cognitive functioning [2]. Reduced cognitive performance is one of the core features of schizophrenia and an important predictor of outcome [3]. Several studies have shown neurocognitive deficits already in patients with a so-called at-risk mental state (ARMS) for psychosis [4]. Furthermore, it has been found that ARMS patients with later conversion to psychosis performed worse at baseline in tests measuring attention/vigilance, speed of processing, verbal and visual learning, and current and premorbid IQ compared with patients who did not convert [4]. Consequently, several studies have shown that the prediction of transition to psychosis can be improved by including neurocognitive performance measures into multivariable risk prediction models [4–8].

Cognitive performance is not only dependent on different stages of psychotic disorders, but also on sex. In healthy controls (HC), it is well established that women tend to perform better than men in tasks measuring verbal abilities (*d* = 0.24; for meta-analysis, see reference [9]), whereas men tend to outperform women on visual–spatial tasks (*d* = 0.45; for meta-analysis, see [9]) [10–12]. Most studies indicate that these differences are also maintained in patients with schizophrenic psychoses (for reviews, see references [1,2]). Specifically, many studies have shown that women diagnosed with schizophrenia have a better performance in verbal learning and memory [1,13,14]. The female advantage in verbal domains has also been found in patients with first-episode psychosis (FEP), while men showed a better performance in tests of reaction time, visual memory, and executive functions [1,10].

The impact of sex on cognitive functioning in ARMS has received considerable attention in the literature in recent years. A meta-regression analysis based on 19 studies assessing neuropsychological performance in 1,188 ARMS patients (women, *n* = 523; 44%) and 1,029 HC (women, *n* = 464; 45%) showed a trend-level significance effect of sex on cognitive performance, with females performing relatively better than males [15]. Our own group investigated sex differences in cognitive functioning in 118 ARMS patients (women, *n* = 45; 38%), 88 FEP patients (women, *n* = 32; 36%), and 86 HC (women, *n* = 41; 47%) [10]. Women performed better in the domain of verbal learning and memory whereas men showed a shorter reaction time during the working memory task across all groups. However, these differences did not withstand correction for multiple testing. Taken together, existing studies indicate that female patients with psychotic disorders or being at clinical high risk for psychosis do not perform better than males over and above what we see in HC.

To the best of our knowledge, the present study is the first to investigate sex differences in cognitive functioning in a large multinational sample of ARMS patients by using an extended neuropsychological battery and a healthy comparison group. The goal of the study was to elucidate whether sex differences in cognitive functioning differ between ARMS and HC subjects. Based on the evidence above and our own findings, we expected a better performance of women in the domain of verbal learning and memory irrespective of group.

## Methods

### Setting and recruitment

The neuropsychological data analyzed in this study were collected within the EUropean Gene–Environment Interactions (EU-GEI) study, which aims to identify the interactive genetic, clinical, and environmental determinants of schizophrenia [16]. EU-GEI is a naturalistic prospective multicenter study that consisted of a baseline and up to three follow-up time points (at 6 months, 12 months, and 24 months). Data were collected from May 1, 2010 to August 6, 2015. For the current analyses, only baseline data, that is, at intake into the study, were used.

ARMS participants were recruited from 11 Early Detection and Intervention Centers (London, Amsterdam, The Hague, Vienna, Basel, Cologne, Copenhagen, Paris, Barcelona, Melbourne, Saõ Paulo). They were referred to the EU-GEI study by primary health care services, mental health professionals, or themselves or their families.

Control participants were recruited by four of the above-mentioned centers: the Institute of Psychiatry, Psychology, and Neuroscience (IoPPN) in London, the Personal Assessment and Crisis Evaluation Clinic in Melbourne, and the Amsterdam Medical Center and Parnassia, The Hague. They were approached by telephone and through advertisements at educational institutes. In Melbourne, controls were additionally approached at community centers/noticeboards and advertised via online platforms. Controls were matched to the ARMS patients in terms of age, sex, migrant, and ethnic status. All participants were screened with an inclusion/exclusion checklist (see below).

The protocol of the EU-GEI study was approved by the institutional review boards of all study sites. EU-GEI was conducted in accordance with the Declaration of Helsinki. The Medical Ethics Committees of all participating sites approved the study protocol.

### Inclusion and exclusion criteria

Inclusion criteria for ARMS patients were: aged 14–45 (most of them were between 18 and 35 years); being at-risk for psychosis as defined by the comprehensive assessment of at-risk mental state (CAARMS) [17]; adequate language skills corresponding to each center; and consent to study participation. The exclusion criteria were: prior experience of a psychotic episode of more than 1-week as determined by the CAARMS [17] and Structural Clinical Interview for Diagnostic and Statistical Manual of Mental Disorders (DSM Disorders (SCID)) [18]; previous treatment with an antipsychotic for a psychotic episode; and IQ < 60.

Inclusion criteria for controls were: aged 18–35; adequate language skills local to each center; no evidence of current or past psychosis (including treatment with antipsychotic medication). Exclusion criteria for controls were similar to those for ARMS participants. Additionally, controls were excluded if they met the criteria for an ARMS status as defined by the CAARMS [17].

### Detection procedure

The CAARMS was used to identify ARMS patients [17]. The CAARMS is a semi-structured interview that encompasses psychotic symptoms and a range of other psychopathological symptoms present during the psychosis prodrome. Individuals were classified as being in an ARMS for psychosis if they met at least one of the following risk criteria: (i) attenuated psychotic symptoms (psychotic symptoms subthreshold either in intensity or frequency); (ii) brief limited psychotic symptoms (recent episode of brief psychotic symptoms that spontaneously resolved within 1 week); or (iii) vulnerability group (a first-degree relative with a psychotic disorder or a diagnosis of a schizotypal personality disorder in combination with a significant drop in functioning). The full criteria can be found elsewhere [17].

### Assessment of sociodemographic and clinical characteristics

Sociodemographic characteristics (e.g. age, sex, ethnicity) were obtained using the modified Medical Research Council sociodemographic schedule [19]. Current cannabis frequency was assessed with the modified version of the Cannabis Experience Questionnaire [20]. Data on comorbid affective and anxiety disorders were assessed with the SCID [18]. Psychiatric medication (i.e., use of antipsychotics, antidepressants, and sedatives) was obtained using a medical history questionnaire, designed by the EU-GEI group. The general level of functioning was assessed with the modified version of the Global Assessment of Functioning (GAF) scale [21].

### Classification and assessment of neuropsychology

Neuropsychological performance of each participant was assessed by trained psychiatrists, psychologists, and research assistants. The neuropsychological tests covered the following seven domains: attention/vigilance, reasoning/problem solving, speed of processing, verbal learning, working memory, social cognition, and current IQ. Test scores were assigned to cognitive domains in accordance with Measurement and Treatment Research to Improve Cognition in Schizophrenia (MATRICS) Consensus Cognitive Battery (MCCB) [22]. Tests that are not part of the MCCB were assigned to domains according to their most commonly used function. The following measures were used to cover the cognitive domains of interest:

- Attention/vigilance: Digit Span Forward subtest of the Wechsler Adult Intelligence Scale-third edition (WAIS-III) [23];
- Reasoning/problem solving: Beads Task [24];
- Speed of processing: Digit Symbol Test of the WAIS-III and the Trail-Making Test parts A and B [25];
- Verbal Learning: Rey Auditory Verbal Learning Test [26];
- Working memory: Digit Span Backwards and Arithmetic subtests of the WAIS-III [23];
- Social cognition: Degraded Affect Recognition Task [27] and the Benton Facial Recognition Test [28]; and
- Current IQ: Block Design total raw score, the information total raw score and the estimate of the total IQ of the shortened WAIS-III [23,29].

### Assessment of psychopathology

The Brief Psychiatric Rating Scale expanded version (BPRS-E) [30] was used to assess psychopathology. Sex differences were investigated using the BPRS total score and the following subscales: BPRS positive symptoms and BPRS negative symptoms [31].

### Statistical analyses

All statistical analyses were performed using R [32]. Because observations were nonindependent, that is, observations from the same center were more similar than observations from different centers, sex differences were analyzed using linear mixed effects models including sex and group (ARMS, HC) as a fixed effects factors and randomly varying intercepts per center to account for the clustering in the data. Linear mixed effects models were applied to evaluate the main effects of sex and group (ARMS, HC) as well as their interactions on cognitive functioning. Dependent variables were *z*-transformed before inclusion to models and sex was included as a binary variable with 0 and 1 describing men and women, respectively. Thus, the regression coefficient for sex described the standardized mean difference (SMD) of women compared with men. The results are presented with and without correction for multiple testing. We used the false discovery rate procedure to adjust *p*-values for multiple testing [33].

## Results

### Sample description

The sample of the present study consisted of 343 ARMS patients (185 men, 158 women) and 67 HC subjects (34 men, 33 women). Sociodemographic and clinical characteristics of our sample are presented in Table 1. Cannabis use was more frequent in male ARMS patients than female ARMS patients (30.51% vs. 18.46% used cannabis at least a few times per year). With regard to comorbid affective and anxiety disorders, female ARMS patients showed more often a current anxiety disorder as well as posttraumatic stress disorders (PTSD) compared with male ARMS patients. There were no significant sex differences regarding any current affective disorder (i.e., current depressive, manic, or hypomanic episode and dysthymic disorder), neither for ARMS nor for HC. With regard to psychopathology, male ARMS patients showed significantly more severe BPRS “negative symptoms” (*p* = 0.006) than female ARMS patients. There were no sex differences in ARMS and HC with regard to age, years of education, current psychiatric medication, global functioning, BPRS “positive symptoms” and BPRS “total score.”

**Table 1.** Sociodemographic and clinical sample characteristics

	ARMS					HC				
	All	Men	Women	*p* value	*N*	All	Men	Women	*p* value	*N*
	(*n* = 343)	(*n* = 185)	(*n* = 158)			(*n* = 67)	(*n* = 34)	(*n* = 33)		
Age	22.4 (4.91)	22.7 (5.08)	22.1 (4.70)	0.210	343	22.9 (4.09)	23.0 (4.09)	22.7 (4.15)	0.720	67
Ethnicity				0.481	342				0.009^**^	67
White	245 (71.6%)	136 (73.5%)	109 (69.4%)			42 (62.7%)	23 (67.6%)	19 (57.6%)		
Black	34 (9.94%)	18 (9.73%)	16 (10.2%)			10 (14.9%)	8 (23.5%)	2 (6.06%)		
Mixed	28 (8.19%)	16 (8.65%)	12 (7.64%)			6 (8.96%)	0 (0.0%)	6 (18.2%)		
Asian	11 (3.22%)	6 (3.24%)	5 (3.18%)			9 (13.4%)	3 (8.82%)	6 (18.2%)		
North African	12 (3.51%)	6 (3.24%)	6 (3.82%)			0 (0.0%)	0 (0.0%)	0 (0.0%)		
Other	12 (3.51%)	3 (1.62%)	9 (5.73%)			0 (0.0%)	0 (0.0%)	0 (0.0%)		
Years of education	14.4 (3.07)	14.4 (3.28)	14.4 (2.83)	0.989	302	16.1 (2.79)	16.6 (2.97)	15.6 (2.55)	0.169	65
Cannabis current frequency				0.029^*^	334				0.081	67
None	247 (74.0%)	121 (68.4%)	126 (80.3%)			49 (73.1%)	22 (64.7%)	27 (81.8%)		
Only once or twice	4 (1.20%)	2 (1.13%)	2 (1.27%)			1 (1.49%)	0 (0.0%)	1 (3.03%)		
A few times each year	17 (5.09%)	7 (3.95%)	10 (6.37%)			4 (5.97%)	4 (11.8%)	0 (0.0%)		
A few times each month	17 (5.09%)	12 (6.78%)	5 (3.18%)			7 (10.4%)	4 (11.8%)	3 (9.09%)		
(More than) once a week	11 (3.29%)	9 (5.08%)	2 (1.27%)			3 (4.48%)	1 (2.94%)	2 (6.06%)		
Every day	38 (11.4%)	26 (14.7%)	12 (7.64%)			3 (4.48%)	3 (8.82%)	0 (0.0%)		
Antipsychotics currently	30 (11.8%)	15 (11.6%)	15 (11.9%)	1.000	255	0 (0.0%)	0 (0.0%)	0 (0.0%)		56
Antidepressants currently	82 (32.2%)	39 (30.2%)	43 (34.1%)	0.595	255	2 (3.57%)	0 (0.0%)	2 (7.14%)	0.491	56
Sedatives currently	15 (5.88%)	7 (5.43%)	8 (6.35%)	0.963	255	1 (1.79%)	0 (0.0%)	1 (3.57%)	1.000	56
Current affective disorder	127 (37.0%)	60 (32.4%)	67 (42.4%)	0.073	343	0 (0.0%)	0 (0.0%)	0 (0.0%)		67
Current anxiety disorder	166 (48.4%)	73 (39.5%)	93 (58.9%)	0.001^**^	343	5 (7.46%)	2 (5.88%)	3 (9.09%)	0.673	67
Current OCD	29 (9.70%)	16 (10.1%)	13 (9.29%)	0.975	299	0 (0.0%)	0 (0.0%)	0 (0.0%)		53
Current PTSD	34 (10.6%)	11 (6.40%)	23 (15.4%)	0.015^*^	321	0 (0.0%)	0 (0.0%)	0 (0.0%)		65
GAF disability, impairment	55.5 (12.3)	55.8 (12.4)	55.1 (12.1)	0.584	331	85.0 (8.98)	85.2 (8.15)	84.7 (9.92)	0.819	66
BPRS positive symptoms	7.48 (3.17)	7.67 (3.28)	7.27 (3.03)	0.254	323	3.17 (0.53)	3.13 (0.43)	3.21 (0.63)	0.550	59
BPRS negative symptoms	5.05 (2.40)	5.38 (2.65)	4.66 (2.02)	0.006^**^	324	3.00 (0.00)	3.00 (0.00)	3.00 (0.00)	0.325	59
BPRS total score	43.6 (10.2)	44.1 (10.6)	43.0 (9.67)	0.361	324	25.4 (2.61)	25.3 (2.24)	25.6 (3.01)	0.618	59

Abbreviations: ARMS, at-risk mental state; BPRS, Brief Psychiatric Rating Scale; GAF, Global Assessment of Functioning; HC, healthy controls; OCD, obsessive–compulsive disorder; PTSD, post-traumatic stress disorder.

Continuous variables are described by means and standard deviation in brackets.

* *p* < 0.05.

** *p* < 0.01.

### Effects of sex and diagnostic group on cognitive functioning

**Figure 1.**
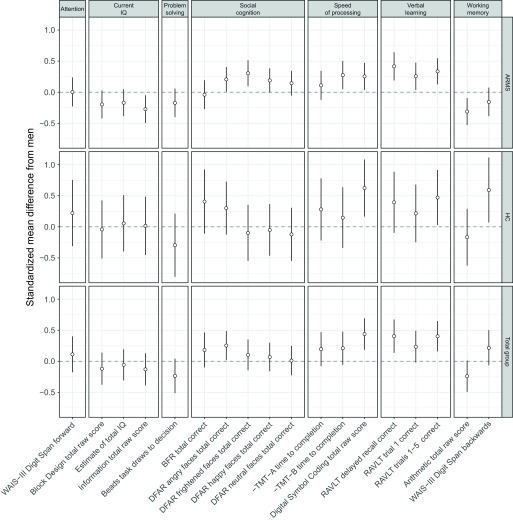
Cognitive performance of women compared with men in at-risk mental state for psychosis individuals and healthy controls. The dotted horizontal line at zero represents the performance of men. Differences are expressed in units of standard deviation and are significant if the 95% confidence interval (vertical line) does not overlap with zero. Variables with a minus sign were reversed so that positive scores always represent good performance. Abbreviations: RAVLT, Rey Auditory Verbal Learning Test; TMT, Trail Making Test; WAIS, Wechsler Adult Intelligence Scale.

Means and standard deviations (*SD*) of the total group, ARMS, and HC are presented in Table 2. Table 3 shows the results of the mixed effects models using neurocognitive performance as the continuous dependent variable and sex as well as group (ARMS, HC) as fixed effects factors. SMDs of the neuropsychological measures are additionally presented in Figure 1.

**Table 2.** Means and standard deviations of neuropsychological test data in ARMS patients and HC

	Total group			ARMS			HC		
	All	Men	Women	All	Men	Women	All	Men	Women
	(*n* = 410)	(*n* = 219)	(*n* = 191)	(*n* = 343)	(*n* = 185)	(*n* = 158)	(*n* = 67)	(*n* = 34)	(*n* = 33)
Attention									
WAIS-III Digit Span Forward	9.69 (2.26)	9.66 (2.19)	9.73 (2.34)	9.51 (2.20)	9.50 (2.16)	9.51 (2.24)	10.7 (2.35)	10.5 (2.18)	11.0 (2.57)
Current IQ									
Block Design total raw score	43.8 (15.2)	44.8 (15.2)	42.7 (15.3)	42.7 (15.5)	43.8 (15.1)	41.4 (15.8)	49.3 (12.9)	49.7 (14.8)	48.9 (11.0)
Estimate of total IQ	101 (17.9)	102 (18.7)	100 (17.0)	98.6 (16.8)	99.9 (17.6)	97.2 (15.9)	113 (18.1)	112 (20.5)	113 (15.7)
Information total raw score	16.9 (6.56)	17.5 (6.56)	16.2 (6.51)	16.3 (6.73)	17.1 (6.71)	15.5 (6.68)	19.4 (4.98)	19.4 (5.47)	19.4 (4.52)
Problem solving									
Beads task draws to decision	6.62 (4.63)	6.90 (5.06)	6.31 (4.11)	6.54 (4.70)	6.74 (5.20)	6.33 (4.09)	7.02 (4.29)	7.73 (4.21)	6.22 (4.32)
Social cognition									
BFR total correct	22.3 (2.33)	22.3 (2.24)	22.3 (2.43)	22.2 (2.32)	22.3 (2.24)	22.2 (2.42)	22.6 (2.36)	22.1 (2.31)	23.1 (2.36)
DFAR angry faces total correct	10.2 (4.90)	9.67 (4.90)	10.7 (4.84)	10.5 (4.71)	9.99 (4.79)	11.0 (4.58)	8.61 (5.53)	7.94 (5.23)	9.30 (5.82)
DFAR frightened faces total correct	8.59 (4.25)	8.17 (4.21)	9.07 (4.26)	8.80 (4.09)	8.26 (4.09)	9.43 (4.02)	7.50 (4.89)	7.68 (4.85)	7.31 (5.01)
DFAR happy faces total correct	12.8 (5.08)	12.5 (5.23)	13.1 (4.89)	13.1 (4.78)	12.7 (5.07)	13.5 (4.38)	11.3 (6.23)	11.4 (6.01)	11.1 (6.54)
DFAR neutral faces total correct	11.4 (4.84)	11.1 (4.95)	11.6 (4.72)	11.6 (4.57)	11.3 (4.76)	12.0 (4.31)	10.2 (5.97)	10.6 (5.92)	9.81 (6.09)
Speed of processing									
Digital Symbol Coding total raw score	73.1 (16.1)	70.2 (16.6)	76.4 (15.0)	71.7 (15.8)	69.2 (16.6)	74.5 (14.4)	80.1 (15.9)	75.2 (15.9)	85.0 (14.6)
TMT-A time to completion	29.6 (12.3)	30.7 (13.9)	28.4 (10.1)	30.2 (12.2)	31.2 (13.6)	29.1 (10.3)	26.6 (12.8)	28.1 (15.6)	25.0 (8.62)
TMT-B time to completion	70.3 (29.4)	74.6 (30.8)	65.7 (27.2)	73.2 (30.4)	77.9 (31.6)	68.0 (28.2)	56.3 (18.8)	58.0 (19.5)	54.4 (18.2)
Verbal learning									
RAVLT delayed recall correct	10.7 (3.14)	10.2 (3.31)	11.4 (2.79)	10.6 (3.05)	10.0 (3.21)	11.2 (2.72)	11.4 (3.48)	10.9 (3.76)	12.0 (3.09)
RAVLT trial 1 correct	6.82 (2.02)	6.58 (1.93)	7.09 (2.09)	6.69 (1.99)	6.45 (1.86)	6.98 (2.11)	7.43 (2.04)	7.25 (2.14)	7.64 (1.93)
RAVLT trials 1–5 correct	52.2 (9.91)	50.6 (9.68)	54.0 (9.88)	51.3 (9.98)	49.9 (9.64)	53.1 (10.1)	56.3 (8.50)	54.1 (9.20)	58.7 (7.01)
Working memory									
Arithmetic total raw score	13.5 (4.76)	14.1 (4.68)	12.8 (4.76)	13.1 (4.70)	13.8 (4.64)	12.3 (4.65)	15.5 (4.58)	15.9 (4.60)	15.1 (4.60)
WAIS-III Digit Span Backwards	6.73 (2.27)	6.74 (2.28)	6.71 (2.27)	6.60 (2.19)	6.72 (2.30)	6.46 (2.06)	7.44 (2.58)	6.87 (2.21)	8.17 (2.87)

Abbreviations: ARMS, at-risk mental state; BFR, Benton Facial Recognition Test; DFAR, Degraded Facial Affect Recognition Task; HC, healthy controls; RAVLT, Rey Auditory Verbal Learning Test; TMT, Trail Making Test; WAIS, Wechsler Adult Intelligence Scale.

**Table 3.** *p* values and coefficients of fixed effects of mixed effects models

	Group			Sex			Group × sex		
	*p* value	*p* value corr^a^	Coef	*p* value	*p* value corr^a^	Coef	*p* value	*p* value corr^a^	Coef
Attention									
WAIS-III Digit Span Forward	<0.001^***^	0.002^**^	0.55	0.441	0.530	0.11	0.467	0.782	0.22
Current IQ									
Block Design total raw score	<0.001^***^	<0.001^***^	0.59	0.364	0.505	−0.12	0.561	0.782	0.15
Estimate of total IQ	<0.001^***^	<0.001^***^	0.88	0.658	0.697	−0.06	0.385	0.782	0.22
Information total raw score	<0.001^***^	<0.001^***^	0.61	0.329	0.493	−0.13	0.277	0.774	0.29
Problem solving									
Beads task draws to decision	0.331	0.426	0.15	0.098	0.252	−0.23	0.659	0.782	−0.13
Social cognition									
BFR total correct	0.143	0.198	0.22	0.201	0.329	0.18	0.124	0.705	0.45
DFAR angry faces total correct	0.435	0.489	−0.10	0.034^*^	0.151	0.25	0.695	0.782	0.09
DFAR frightened faces total correct	0.421	0.489	−0.11	0.409	0.525	0.10	0.112	0.705	−0.40
DFAR happy faces total correct	0.531	0.563	−0.08	0.547	0.615	0.07	0.301	0.774	−0.24
DFAR neutral faces total correct	0.922	0.922	−0.01	0.922	0.922	0.01	0.266	0.774	−0.27
Speed of processing									
Digital Symbol Coding total raw score	<0.001^***^	<0.001^***^	0.63	<0.001^***^	0.011^*^	0.44	0.157	0.705	0.37
TMT-A time to completion	0.017^*^	0.028^*^	−0.36	0.162	0.291	−0.20	0.549	0.782	−0.17
TMT-B time to completion	<0.001^***^	<0.001^***^	−0.65	0.122	0.261	−0.21	0.641	0.782	0.13
Verbal learning									
RAVLT delayed recall correct	0.026^*^	0.040^*^	0.33	0.003^**^	0.019^*^	0.41	0.936	0.936	−0.02
RAVLT trial 1 correct	0.016^*^	0.028^*^	0.34	0.069	0.206	0.24	0.874	0.926	−0.04
RAVLT trials 1–5 correct	<0.001^***^	<0.001^***^	0.49	0.001^**^	0.011^*^	0.40	0.593	0.782	0.13
Working memory									
Arithmetic total raw score	<0.001^***^	<0.001^***^	0.60	0.064	0.206	−0.24	0.580	0.782	0.14
WAIS-III Digit Span Backwards	<0.001^***^	0.002^**^	0.51	0.130	0.261	0.22	0.011^*^	0.197	0.75

Abbreviations: BFR, Benton Facial Recognition Test; coef, *y*-standardized regression coefficients of fixed effects; DFAR, Degraded Facial Affect Recognition Task; RAVLT, Rey Auditory Verbal Learning Test; TMT, Trail Making Test; WAIS, Wechsler Adult Intelligence Scale.

a Corrected for multiple testing using Benjamini–Hochberg method.

* *p* < 0.05.

** *p* < 0.01.

^***^ *p* < 0.001.

In the combined sample of ARMS and HC, women recognized more angry faces in the “Degraded Faces Affect Recognition” social cognition task (*p* = 0.034, *b* = 0.25), performed better in the “Digital Symbol Coding” speed of processing task (*p* ≤ 0.001, *b* = 0.44) of the WAIS-III, and remembered more words in the “Rey Auditory Verbal Learning Test (RAVLT) delayed recall” (*p* = 0.003, *b* = 0.41) and “RAVLT trials 1 to 5” (*p* = 0.001, *b* = 0.40) than men. However, after correction for multiple testing, only the differences in “Digital Symbol Coding” and the RAVLT measures remained statistically significant.

Effects of diagnostic group are presented in Table 3. ARMS patients performed significantly worse in all cognitive performance scores, except in all scores of the problem solving and social cognition tasks.

There was one statistically significant interaction between sex and group (ARMS, HC) on the “WAIS-III Digit Span Backwards” working memory task (*p* = 0.011), which was due to a significantly better performance of female HC compared with male HC (*p* < 0.026, *b* = −0.59) and a nonsignificantly worse performance of female ARMS patients compared with male ARMS patients (*p* = 0.186, *b* = 0.16). However, this sex × group interaction was no longer significant after correction for multiple testing.

The results did not change, when age or frequent cannabis use (i.e., at least several times per week) were included as covariates.

## Discussion

To the best of our knowledge, this is the first study investigating sex-related neurocognitive performance differences in a multinational ARMS sample of this size, using a comprehensive neuropsychological battery and a healthy comparison group. In line with our hypotheses, women showed superior performance in the domain of verbal learning and memory independent of whether they were ARMS patients or HC. Furthermore, women outperformed men on measures of speed of processing (i.e., Digital Symbol Coding total raw score) and social cognition (i.e., Degraded Facial Affect Recognition Task (DFAR) angry faces total correct), whereas men outperformed women on a trend-wise level on a task of working memory (i.e., arithmetic total raw score). Additionally, our results show that ARMS patients displayed alterations in attention, current IQ, speed of processing, verbal learning, and working memory compared with HC. However, we will not discuss this aspect any further since it is not the focal point of the present study.

Finally, we found a sex × group interaction effect on working memory (i.e., WAIS-III Digit Span Backwards), which was due to a significantly better performance of female HC compared with male HC and a nonsignificantly better performance of male ARMS patients compared with female ARMS patients. However, only sex differences in the total group in speed of processing and verbal learning remained significant after correction for multiple testing.

With regard to verbal learning and memory, our finding that the female advantage is equally present in ARMS patients as in HC is in line with previous research [1,15]. Furthermore, it corroborates the findings of an earlier study of our own group that reported no interaction effect between diagnostic group (i.e., ARMS, FEP, HC) and verbal learning and memory [10].

Regarding processing speed, our finding that women perform better than men is also consistent with earlier findings from the general population [34,35] and patients with schizophrenia [36,37]. To the best of our knowledge, this is the first study examining sex differences in ARMS and healthy subjects by using well-established tests to evaluate processing speed (i.e., Trail Making Test, WAIS-III Digit Symbol subtest). A previous study has investigated sex-related cognitive performance differences in ARMS, FEP and HC but did not include tests specifically measuring processing speed [10]. However, the authors found a shorter reaction time for men in the working memory task independent of diagnostic group. They explain the findings by a superior working memory performance rather than generally enhanced processing speed in men as no sex differences in reaction time during the Continuous Performance Test and the Go/No-Go subtest of the Test of Attentional Performance (TAP) were detected, while maintaining a comparable overall working memory performance level [10].

A strength of our study is that we examined sex differences with well-established tests using the classification of the MATRICS panel [22,38]. As the MCCB domains are well known in schizophrenia research, this may help future studies to compare sex-related cognitive performance differences in ARMS and schizophrenic patients. Furthermore, this is the first study to investigate sex differences in cognitive functioning in an ARMS sample of this size.

However, there are some limitations to the present study that need to be acknowledged. Our neuropsychological test battery was originally selected to identify genetic and environmental interactions in psychosis and not specifically to detect sex differences. Accordingly, the test battery did not include other sensitive tasks to detect sex differences such as visuo-spatial tasks. Additionally, the domain of visual learning in the MATRICS consensus battery was not covered. Furthermore, our control group was rather small in comparison to the ARMS group, which reduced the statistical power to detect interaction effects between sex and group. Finally, it is important to note that sex-related cognitive performance differences depend on a wide variety of conditions, for example, the severity of symptoms and especially the fluctuation of estrogen levels during the menstrual cycle in women (for review, see reference [1]). There is evidence that high levels of estrogen at the mid-luteal point are associated with better verbal memory and diminished spatial ability [39]. Thus, it is possible that some effects would have changed if we had measured women at a specific time point during their monthly cycle. Unfortunately, in our study no assessment of the time point during the monthly cycle was performed.

Taken together, our findings indicate that sex differences in cognitive functioning in ARMS patients are very similar to those seen in the general population and in schizophrenia patients.

## Appendix

### EU-GEI High Risk Study Group—Author List

Philip McGuire^2^, Lucia R. Valmaggia^3^, Matthew J. Kempton^2^, Maria Calem^2^, Stefania Tognin^2^, Gemma Modinos^2^, Lieuwe de Haan^4,7^, Mark van der Gaag^8,10^, Eva Velthorst^5,11^, Tamar C. Kraan^6^, Daniella S. van Dam^4^, Nadine Burger^7^, Barnaby Nelson^12,13^, Patrick McGorry^12,13^, G. Paul Amminger^12,13^, Christos Pantelis^14^, Athena Politis^12,13^, Joanne Goodall^12,13^, Anita Riecher-Rössler^1^, Stefan Borgwardt^1^, Charlotte Rapp^1^, Sarah Ittig^1^, Erich Studerus^1^, Renata Smieskova^1^, Rodrigo Bressan^15^, Ary Gadelha^15^, Elisa Brietzke^16^, Graccielle Asevedo^15^, Elson Asevedo^15^, Andre Zugman^15^, Neus Barrantes-Vidal^17^, Tecelli Domínguez-Martínez^18^, Anna Racioppi^19^, Lídia Hinojosa-Marqués^19^, Thomas R. Kwapil^20^, Manel Monsonet^19^, Mathilde Kazes^21^, Claire Daban^21^, Julie Bourgin^21^, Olivier Gay^21^, Célia Mam-Lam-Fook^21^, Marie-Odile Krebs^21^, Dorte Nordholm^22^, Lasse Randers^22^, Kristine Krakauer^22^, Louise Glenthøj^22^, Birte Glenthøj^23^, Merete Nordentoft^22^, Stephan Ruhrmann^24^, Dominika Gebhard^24^, Julia Arnhold^25^, Joachim Klosterkötter^24^, Gabriele Sachs^26^, Iris Lasser^26^, Bernadette Winklbaur^26^, Philippe A. Delespaul^27,28^, Bart P. Rutten^29^, and Jim van Os^29,30^.

### Affiliations

^1^University Psychiatric Hospital, CH-4002 Basel, Switzerland; ^2^Department of Psychosis Studies, Institute of Psychiatry, Psychology & Neuroscience, King’s College London, London SE5 8AF, United Kingdom; ^3^Department of Psychology, Institute of Psychiatry, Psychology & Neuroscience, King’s College London, London SE5 8AF, United Kingdom; ^4^Amsterdam UMC, University of Amsterdam, Psychiatry, Department Early Psychosis, Amsterdam, The Netherlands; ^5^Department of Psychiatry and Seaver Center for Research and Treatment, Icahn School of Medicine at Mount Sinai, New York, USA; ^6^Mental Health Institute Arkin, Amsterdam, The Netherlands; ^7^Arkin Amsterdam; ^8^VU University, Faculty of Behavioral and Movement Sciences, Department of Clinical Psychology and Amsterdam Public Mental Health Research Institute, 1081 BT Amsterdam, The Netherlands; ^9^Mental Health Institute Noord-Holland Noord, Hoorn, The Netherlands; ^10^Parnassia Psychiatric Institute, Department of Psychosis Research, 2512 HN The Hague, The Netherlands; ^11^Early Psychosis Section, Department of Psychiatry, Academic Medical Centre, University of Amsterdam, Amsterdam, The Netherlands; ^12^Orygen, The National Centre of Excellence in Youth Mental Health, University of Melbourne, Melbourne, Australia; ^13^Centre for Youth Mental Health, University of Melbourne, Parkville, Victoria 485 3052, Australia; ^14^Melbourne Neuropsychiatry Centre, The University of Melbourne, Melbourne, Australia; ^15^LiNC—Lab Interdisciplinar Neurociências Clínicas, Depto Psiquiatria, Escola Paulista de Medicina, Universidade Federal de São Paulo—UNIFESP, São Paulo, Brazil; ^16^Depto Psiquiatria, Escola Paulista de Medicina, Universidade Federal de São Paulo—UNIFESP, São Paulo, Brazil; ^17^Departament de Psicologia Clínica i de la Salut (Universitat Autònoma de Barcelona), Fundació Sanitària Sant Pere Claver (Spain), Spanish Mental Health Research Network (CIBERSAM), Spain; ^18^CONACYT-Dirección de Investigaciones Epidemiológicas y Psicosociales, Instituto Nacional de Psiquiatría Ramón de la Fuente Muñiz, Mexico; ^19^Departament de Psicologia Clínica i de la Salut (Universitat Autònoma de Barcelona), Bellaterra, Spain; ^20^Department of Psychology, University of Illinois at Urbana-Champaign, Urbana, Illinois, USA; ^21^University Paris Descartes, GHU Paris Sainte-Anne, C’JAAD, Service Hospitalo-Universitaire, Inserm 1266, Institut de Psychiatrie (CNRS 3557) Paris, France; ^22^Mental Health Center Copenhagen and Center for Clinical Intervention and Neuropsychiatric Schizophrenia Research, CINS, Mental Health Center Glostrup, Mental Health Services in the Capital Region of Copenhagen, University of Copenhagen, Copenhagen, Denmark; ^23^Centre for Neuropsychiatric Schizophrenia Research (CNSR) & Centre for Clinical Intervention and Neuropsychiatric Schizophrenia Research (CINS), Mental Health Centre Glostrup, University of Copenhagen, Glostrup, Denmark; ^24^Department of Psychiatry and Psychotherapy, University of Cologne, Cologne, Germany; ^25^Psyberlin, Berlin, Germany; ^26^Medical University of Vienna, Department of Psychiatry and Psychotherapy, Vienna, Austria; ^27^Department of Psychiatry and Neuropsychology, School for Mental Health and Neuroscience, Maastricht University Medical Centre, 6200 MD 464 Maastricht, The Netherlands; ^28^Mondriaan Mental Health Trust, 4436 CX Heerlen, The Netherlands; ^29^Medical University of Vienna, Department of Psychiatry and Psychotherapy; ^30^Department of Psychosis Studies, Institute of Psychiatry, Psychology & Neuroscience, King’s College London, London SE5 8AF, United Kingdom**.**
